# Anatomical Variation of the Maxillary Sinus in Cone Beam Computed Tomography

**DOI:** 10.1155/2014/707261

**Published:** 2014-02-12

**Authors:** Marcelo Lupion Poleti, Luciana Maria Paes da Silva Ramos Fernandes, Christiano Oliveira-Santos, Ana Lúcia Alvares Capelozza, Luiz Eduardo Montenegro Chinellato, Izabel Regina Fischer Rubira-Bullen

**Affiliations:** ^1^University of North Paraná (UNOPAR), Rua Marselha, 183, Jardim Piza, 86041-140 Londrina, PR, Brazil; ^2^Department of Stomatology, Bauru School of Dentistry, University of São Paulo, Alameda Otávio Pinheiro Brisolla, 9-75, Vila Universitária, 17012-901 Bauru, SP, Brazil; ^3^Department of Morphology, Stomatology and Physiology, Ribeirão Preto School of Dentistry, University of São Paulo, Avenida do Café, s/n, Bairro Monte Alegre, 14040-904 Ribeirão Preto, SP, Brazil

## Abstract

*Purpose*. The aim of this paper is to report a case in which the cone beam computed tomography (CBCT) was important for the confirmation of the presence of maxillary sinus septum and, therefore, the absence of a suspected pathologic process. *Case Description*. A 27-year-old male patient was referred for the assessment of a panoramic radiograph displaying a radiolucent area with radiopaque border located in the apical region of the left upper premolars. The provisional diagnosis was either anatomical variation of the maxillary sinuses or a bony lesion. *Conclusion*. The CBCT was important for an accurate assessment and further confirmation of the presence of maxillary septum, avoiding unnecessary surgical explorations.

## 1. Introduction

Maxillary sinuses (MS) are facial pyramidal cavities with thin walls corresponding to the orbital, alveolar, facial, and infratemporal aspects of the maxilla. The size, shape, and wall thickness of this anatomic structure vary from one individual to another [1]. Some maxillary sinuses present septa, consisting of thin walls of bone inside the sinus, which are variable in thickness, length, and number [2]. According to the literature, the incidence of maxillary sinus septa is between 16 and 58% [1]. Acknowledgement of the anatomic variations of the MS is very important for surgeons, specially prior to surgical procedures, such as insertion of maxillary dental implants or maxillary sinus lifting. MS septa may be more accurately diagnosed using computed tomography (CT) or more recently cone beam computed tomography (CBCT). The information offered by these imaging resources is used to predict sinus volume and the degree of septation, when present [3–5]. This paper reports a case in which the CBCT was important for the confirmation of the presence of maxillary sinus septum and, therefore, the absence of a suspected pathologic process.

## 2. Case Presentation

**Figure 1:**
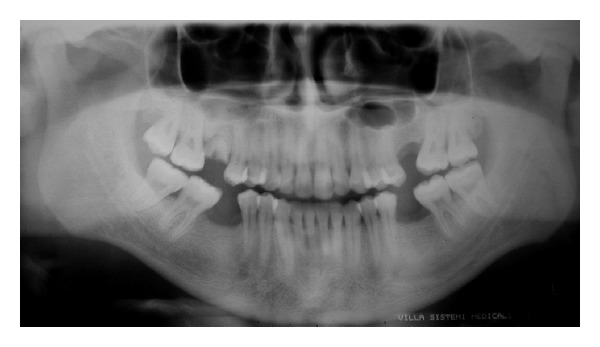
Panoramic radiograph showing radiolucent area surrounded by radiopaque halo located in the periapical region of the upper first and second premolars.

A 27-year-old male patient was referred to our institution for the assessment of a radiolucent area surrounded by radiopaque border located in the periapical regions of upper left first and second premolars (Figure 1).

The intraoral examination showed integrity of the mucosa and no bone expansion was noticed. Pulp vitality test was positive for all teeth in the region. The provisional diagnosis was an anatomic variation of the MS or a bony lesion.

**Figure 2:**
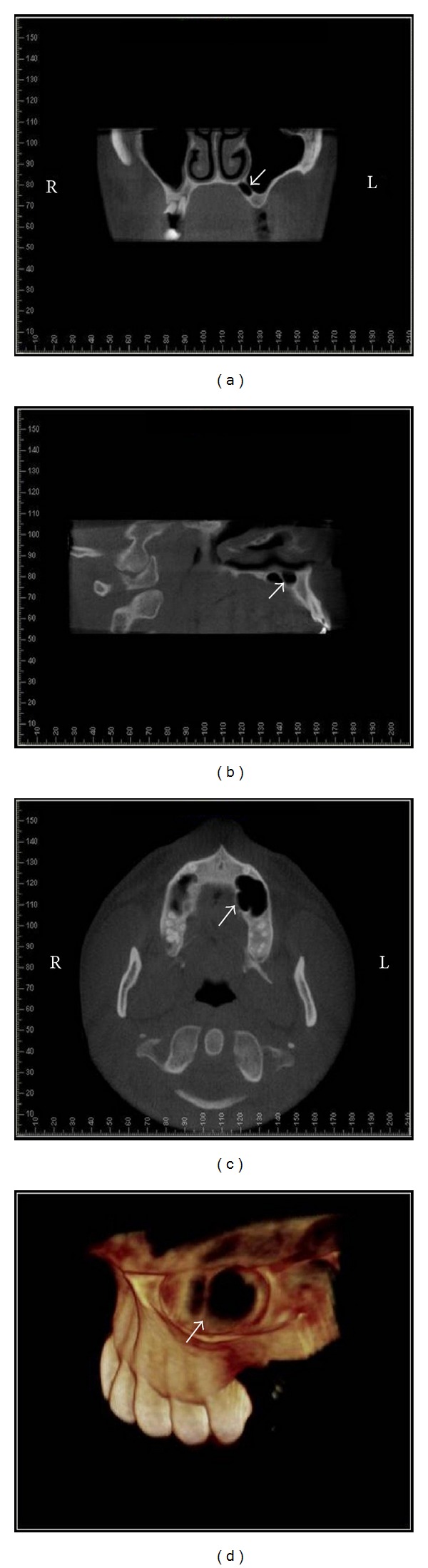
CBCT images: coronal (a), sagittal (b), and axial (c) slices and 3D reconstruction (d) in which the septum and palatine extension maxillary sinus are clearly seen on left side (arrows). R: right side and L: left side.

In order to visualize the region in 3D, CBCT scan was performed (i-CAT Classic/Imaging Sciences, USA), using FOV of 6 cm, 20 seconds, and 0.3 mm voxel. The images were reformatted using *Dolphin* software (Dolphin Imaging & Management Solutions, Patterson Technology, USA). Coronal, sagittal, and axial images showed the presence of maxillary sinus septum and associated with palatine extension of maxillary sinus. 3D reconstructions allowed the volumetric visualization of the anatomic condition (Figure 2).

These tomographic images confirmed the provisional diagnosis of anatomical variation of the MS and the absence of any related pathologic process. The CBCT showed the location and size of the septum as well as its association with a palatine sinus extension. The patient has been informed about the anatomical variation.

## 3. Discussion

Many authors have reported the presence of MS septa and their relevance to surgical procedures. This anatomic variation was first described by Underwood in 1910 [1]. The presence of MS septa can be detected in panoramic radiographs. However, CT and CBCT are definitely the preferred imaging techniques for the assessment of this anatomic variation. Krennmair et al. [4] found that panoramic radiograph can lead to false diagnosis regarding the positive or negative identification of septa in 21.3% of cases. They stated that CT scanning is the preferred imaging method for the detection of the presence (or absence) of sinus septa, since it allows the high-resolution imaging of delicate bony structures. Özyuvaci et al. [5] published a paper about the maxillary sinus lift operation. The authors recommend the use of three-dimensional (3D) assessment of the region using CT and criticize the use of plain radiographs for the estimation of the bone volume and assessment of the anatomic condition of the sinus, considering that the information brought by conventional radiographs is very limited and sometimes not reliable. González-Santana et al. [2] evaluated 60 maxillary sinuses of 30 patients using panoramic radiographs and helical CT scan. The authors founded that 25% of these sinuses had septa. The panoramic radiographs failed to show the septa in some cases. The authors concluded that CT scan is more reliable than the panoramic radiograph for the observation of maxillary septa due to its greater accuracy.

In the present case, the first analysis of the panoramic radiograph led to a concern about the possibility of the radiolucent image to represent a cystic lesion. The image could be suggestive of a maxillary cystic process of odontogenic origin, since the radiolucent area was close to the apical region of the teeth and it presented a surrounding radiopaque border. The clinical examination proved that the teeth were vital and the patient had no pain or swelling. Nevertheless, the radiolucent image as seen on the panoramic radiograph could still represent an odontogenic lesion at an early stage. The CBCT scan was important for the accurate assessment of the region and identification of the sinus septum and palatine extension.

## 4. Conclusion

As a conclusion, this paper highlights the importance of an accurate imaging assessment of the maxillary sinus and its variations, in order to properly differentiate pathologic lesions from an anatomic variation, avoiding unnecessary surgical explorations.
